# Automated Assessment of the Pulmonary Artery-to-Ascending Aorta Ratio in Fetal Cardiac Ultrasound Screening Using Artificial Intelligence

**DOI:** 10.3390/bioengineering11121256

**Published:** 2024-12-12

**Authors:** Rina Aoyama, Masaaki Komatsu, Naoaki Harada, Reina Komatsu, Akira Sakai, Katsuji Takeda, Naoki Teraya, Ken Asada, Syuzo Kaneko, Kazuki Iwamoto, Ryu Matsuoka, Akihiko Sekizawa, Ryuji Hamamoto

**Affiliations:** 1 Division of Medical AI Research and Development, National Cancer Center Research Institute, 5-1-1 Tsukiji, Chuo-ku, Tokyo 104-0045, Japan; 2 Department of Obstetrics and Gynecology, School of Medicine, Showa University, 1-5-8 Hatanodai, Shinagawa-ku, Tokyo 142-8666, Japan; 3 Cancer Translational Research Team, RIKEN Center for Advanced Intelligence Project, 1-4-1 Nihonbashi, Chuo-ku, Tokyo 103-0027, Japan; 4 HLPF Data Analytics Department, Fujitsu Ltd., 1-5 Omiya-cho, Saiwai-ku, Kawasaki 212-0014, Japan; 5 Department of NCC Cancer Science, Biomedical Science and Engineering Track, Graduate School of Medical and Dental Sciences, Institute of Science Tokyo, 1-5-45 Yushima, Bunkyo-ku, Tokyo 113-8510, Japan; 6 Artificial Intelligence Laboratory, Fujitsu Ltd., 4-1-1 Kamikodanaka, Nakahara-ku, Kawasaki 211-8588, Japan

**Keywords:** fetal cardiac ultrasound screening, three-vessel view, PA/Ao ratio, artificial intelligence

## Abstract

The three-vessel view (3VV) is a standardized transverse scanning plane used in fetal cardiac ultrasound screening to measure the absolute and relative diameters of the pulmonary artery (PA), ascending aorta (Ao), and superior vena cava, as required. The PA/Ao ratio is used to support the diagnosis of congenital heart disease (CHD). However, vascular diameters are measured manually by examiners, which causes intra- and interobserver variability in clinical practice. In the present study, we aimed to develop an artificial intelligence-based method for the standardized and quantitative evaluation of 3VV. In total, 315 cases and 20 examiners were included in this study. We used the object-detection software YOLOv7 for the automated extraction of 3VV images and compared three segmentation algorithms: DeepLabv3+, UNet3+, and SegFormer. Using the PA/Ao ratios based on vascular segmentation, YOLOv7 plus UNet3+ yielded the most appropriate classification for normal fetuses and those with CHD. Furthermore, YOLOv7 plus UNet3+ achieved an arithmetic mean value of 0.883 for the area under the receiver operating characteristic curve, which was higher than 0.749 for residents and 0.808 for fellows. Our automated method may support unskilled examiners in performing quantitative and objective assessments of 3VV images during fetal cardiac ultrasound screening.

## 1. Introduction

Congenital heart disease (CHD) is a common condition with an estimated global prevalence of nearly 1.8 cases per 100 live births. Its severity varies widely; some infants spontaneously recover, while the condition disappears immediately after birth in others [1]. Prenatal diagnosis and appropriate risk assessment during the fetal period can allow more careful consideration of the delivery facility and method, allowing infants to be hospitalized immediately after birth without going into shock, thereby increasing the likelihood that they will be in a better condition when they are treated. This is expected to improve short- and long-term prognoses, including surgical outcomes [2,3]. Prenatal counseling is also beneficial for the baby’s family [4,5,6].

Ultrasound imaging is the most important modality used in prenatal screening for CHD. Its non-invasive and real-time nature enables the assessment of fetal cardiac morphology and function. In fetal cardiac ultrasound screening, information obtained from various cross-sections is combined. Several standard transverse scanning planes of the fetal heart can be used, including the four-chamber view (4CV), three-vessel view (3VV), and three-vessel tracheal view (3VTV). The reported sensitivity of 4CV plus the outflow tract is 65.5%, which is superior to the 60.3% sensitivity of 4CV alone [7]. Furthermore, adding 3VTV and its color-flow mapping to 4CV reportedly increases the detection rate to 88.5% [8]. However, the global prenatal diagnostic rate of 40–50% remains insufficient [9,10]. This situation depends on the differences in equipment and skill levels between examiners [11].

Advances in artificial intelligence (AI) have facilitated substantial research in a wide range of medical fields [12,13,14,15]. Hence, AI has been introduced to support unskilled examiners in performing fetal ultrasound assessment [16,17]. Such assessments can follow several approaches, including anatomical structure analysis, biometric parameter estimation, and standard plane detection. The most frequently reported anatomical structures analyzed using AI in fetal ultrasound images are the heart, head, placenta, and amniotic fluid. The standard planes of the fetal heart automatically extracted by AI include the 4CV, 3VV, and left ventricular outflow tract [16]. The most commonly reported fetal evaluation index calculated using AI is the head circumference. The biparietal diameter, abdominal circumference, and femoral length, which are used to estimate fetal weight, have also been evaluated using AI [18]. Time-series information in ultrasound videos can be incorporated into AI models to facilitate the accurate segmentation of cardiac substructures [19,20]. Moreover, several explainable representations have been introduced to improve screening performance in fetal cardiac ultrasound screening [21]. Furthermore, several novel predictive indicators of clinical outcomes have been proposed. For example, the risk of complications in pregnancy may be predicted using the placental volume automatically calculated from three-dimensional ultrasound images during early pregnancy [22], while respiratory disorders in the neonatal period may be predicted using texture analysis of the fetal lungs [23]. Another innovative approach is the quantitative analysis of fetal brain activity via automatic recognition of the changes in fetal facial expressions in four-dimensional ultrasound images [24].

As representative biometric parameters to support the detection of CHD, the ratio of the diameter of the pulmonary artery (PA) to that of the ascending aorta (Ao) (PA/Ao ratio) in 3VV, the cardiothoracic area ratio, and the cardiac axis in 4CV are manually measured, as required [25]. However, the fundamental problem is that the accuracy of the measurements of the abovementioned indices depends on the examiner’s experience and skill level [26]. In this study, we focused on the PA/Ao ratio and aimed to demonstrate an AI-based method for the automated and quantitative evaluation of 3VV in fetal cardiac ultrasound screening.

## 2. Materials and Methods

### 2.1. Data Preparation

A total of 315 women with singleton pregnancies underwent fetal cardiac ultrasound screening at Showa University Hospital (Tokyo, Japan). The data comprised 280 normal cases and 35 CHD cases. The mean (range) gestational age was 21 weeks (18–34 weeks) for normal cases and 23 weeks (19–35 weeks) for CHD cases. The characteristics of the CHD cases are listed in Supplementary Table S1. Most ultrasound data were obtained using fetal echocardiography, while some were obtained under the supervision of a fetal echocardiologist. Videos were acquired using commercially available ultrasonography machines (Voluson^®^ E8 or E10; GE Healthcare, Chicago, IL, USA) equipped with an abdominal 2–6 MHz transducer in accordance with the relevant guidelines [27]. A cardiac preset was used, and the images were magnified until the chest filled at least half to two-thirds of the screen. Each video comprised sequential cross-sections from the level of the stomach through the heart to the vascular arches, mainly in the apical view. For the manual extraction of the 3VV, one obstetrician extracted the appropriate image from the video under the supervision of two experts.

### 2.2. Data Preprocessing and Augmentation

The obstetrician annotated the PA, Ao, and superior vena cava (SVC) in the 3VV pixel-by-pixel, creating accurate labels under the supervision of two experts. The training data were extracted from normal cases. The diversity in the training dataset of 470 images from 270 normal cases was within the acceptable range, while the image quality control was almost consistent with the abovementioned data preparation. We randomly assigned 376 images from 216 normal cases for training and 94 images from 54 normal cases for testing. For CHD cases, 81 images from 35 fetuses were used for testing. The data were assigned for training and validation in a 4:1 ratio, with no overlaps across the training, validation, or testing datasets. For cross-validation, five sets of training and test data were created, such that all normal cases were included in the test data (Supplementary Figure S1).

The object-detection model YOLOv7 was used for automated 3VV extraction to allow comparison with manual 3VV extraction [28]. This model was configured to recognize 18 substructures: the crux, ventricular septum, right atrium, tricuspid valve, right ventricle, left atrium, mitral valve, left ventricle, PA, Ao, SVC, descending aorta, stomach, spine, umbilical vein, inferior vena cava, pulmonary vein, and ductus arteriosus [29]. For automated 3VV extraction, the PA, Ao, and SVC were detected simultaneously with confidence thresholds of 0.001, 0.1, and 0.01, respectively. Image extraction was performed randomly, and a limit of two images per video was extracted for normal cases. The same set of cases was used for both training and test data for manual 3VV extraction. The final dataset consisted of 431 images of normal cases and 102 images of CHD cases obtained using YOLOv7.

Owing to the limited amount of data, we conducted data augmentation, which involved modifying the rotation, brightness, and contrast of the training data. The images were rotated within a range of ±15 degrees. Brightness and contrast were adjusted using the following formula, with the original image as the function src and the output image as the function dst:

(1) dst (I)=saturate_cast (src(I)×α+β).

The values of α and β ranged from 0.7 to 1.3 and −30 to 30, respectively. As a result of the data augmentation, the training data increased 21-fold.

### 2.3. Vascular Segmentation and the PA/Ao Ratio

#### 2.3.1. Vascular Segmentation

To segment the PA/Ao/SVC in the extracted 3VV, we used the following segmentation models: DeepLabv3+ [30], UNet3+ [31], and SegFormer [32]. The hyperparameters for each model were obtained from the literature. ImageNet was used to pretrain DeepLabv3+ and SegFormer. The Dice coefficient was applied to measure the accuracy of the segmentation model based on true positive (TP), false positive (FP), and false negative (FN) values. The Dice coefficient is calculated as follows:

Dice = 2TP/(2TP + FP + FN).(2)

The Dice coefficients ranged between 0 and 1, where values closer to 1 indicate better segmentation performance. To evaluate the accuracy of the three segmentation models, the mean Dice values (mDice) were calculated for each image using the ground-truth labels and inference results.

#### 2.3.2. PA/Ao Ratio

The number of pixels in the annotated labels and the inference results for the vascular diameters of the PA and Ao were counted automatically. The PA/Ao ratio was calculated as follows:

(3) $$PA/Aoratio=\frac{PA}{Ao}$$

where *PA* is the diameter of PA, and *Ao* is the diameter of Ao. *PA* and *Ao* were obtained from the segmented or annotated vascular region, D, using the following procedure: First, let line ℓ be the line that maximizes the length of the line segment perpendicular to the long axis of region D. Then, *PA* and *Ao* were calculated in region D by using the following formula, with vectors $a={\left(x_{1}{\;y}_{1}\right)}^{T}$ and $b={\left(x_{2}{\;y}_{2}\right)}^{T}$ representing two points, A and B, in region D:

(4) $$PA,\;Ao=\underset{\begin{array}{c} a,b\in D,\;a\neq b\\ AB⊥l \end{array}}{\max}\left\|a-b\right\|.$$

**Figure 1:**
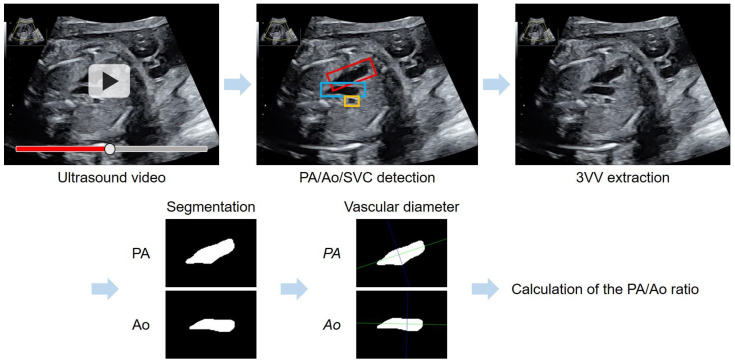
Flowchart of the automated method for the assessment of the PA/Ao ratio in the 3VV. YOLOv7 was applied for an automated 3VV extraction from ultrasound videos; the PA is shown with a red box, Ao with blue, and SVC with yellow. Following vascular segmentation, a purple line maximized the length of the segment perpendicular to the long axis (green line) of each vascular region.

$\left\|a-b\right\|$ represents the distance between vectors a and b. AB⊥l represents the perpendicularity between AB and ℓ. Figure 1 presents a flowchart of the automated method for 3VV extraction using YOLOv7 and PA/Ao-ratio calculation based on vascular segmentation.

**Figure 2:**
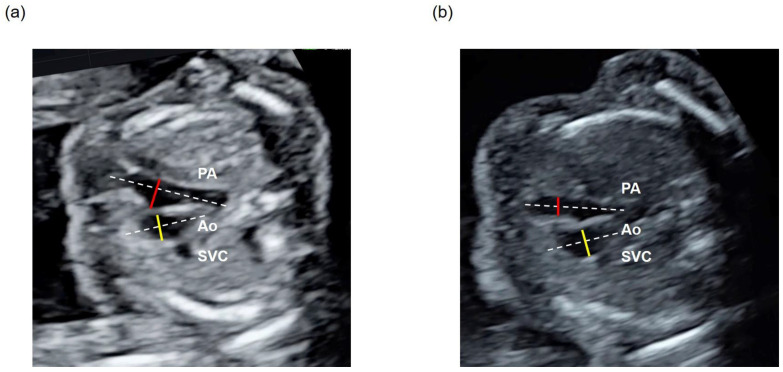
Assessment of the PA/Ao ratio in the 3VV of (**a**) a normal case and (**b**) a patient with CHD with a low PA/Ao ratio. The diameter of the PA is shown using a red line and that of Ao with a yellow line, while the long axis of each vascular region is shown with a white dotted line.

The ±2 standard deviation (SD) range of the PA/Ao ratio obtained from the ground-truth labels was defined as the standard value. These ground-truth labels were annotated in the 3VV of all datasets of the 270 normal cases via manual image extraction. CHD cases were classified into three groups: a low group with a PA/Ao ratio < −2SD, a normal group with a PA/Ao ratio within ±2SD, and a high group with a PA/Ao ratio > +2SD. With reference to the existing literature, all PA/Ao ratios exceeding 3.5 were considered outliers and were changed to 3.5 (Figure 2) [33].

To select the most appropriate method, we first compared the PA/Ao ratios calculated using the ground-truth labels of the normal cases for each method. We subsequently selected the method that showed the best classification performance for the normal and three CHD groups.

### 2.4. Screening-Performance Comparison Study

Twenty examiners (three experts, eight fellows, and nine residents) were enrolled in this screening-performance comparison study. The experts were fetal echocardiologists, the fellows were obstetricians with at least 3 years of experience, while the residents had less than 3 years of experience. Each video in the test dataset, comprising ten videos of ten normal cases and ten videos of ten CHD cases, was rated. Similar to the clinical scenario, examiners manually extracted one image of the 3VV per video, which was suitable for calculating the PA/Ao ratio. Lines were manually drawn on PA and Ao at points that could be considered diameters. Pixel lengths were automatically calculated to assess the PA/Ao ratio calculations (Supplementary Figure S2). In contrast, our automated method simultaneously extracted the 3VV and determined the length of the PA/Ao/SVC. Image segmentation and PA/Ao ratio calculations were performed using the proposed algorithm. To evaluate the performance of fetal cardiac ultrasound screening based on the PA/Ao ratio, receiver operating characteristic (ROC) curve analysis was performed using the absolute value of the calculated PA/Ao ratio (the mean of the standard PA/Ao ratio), taking into account abnormalities above and below the standard value. The arithmetic means of the areas under the ROC curve (AUCs) were compared between the examiners and the proposed method. The higher the AUC value, the better the performance at discriminating between normal and CHD cases.

## 3. Results

### 3.1. Evaluation of Vascular Segmentation

**Figure 3:**
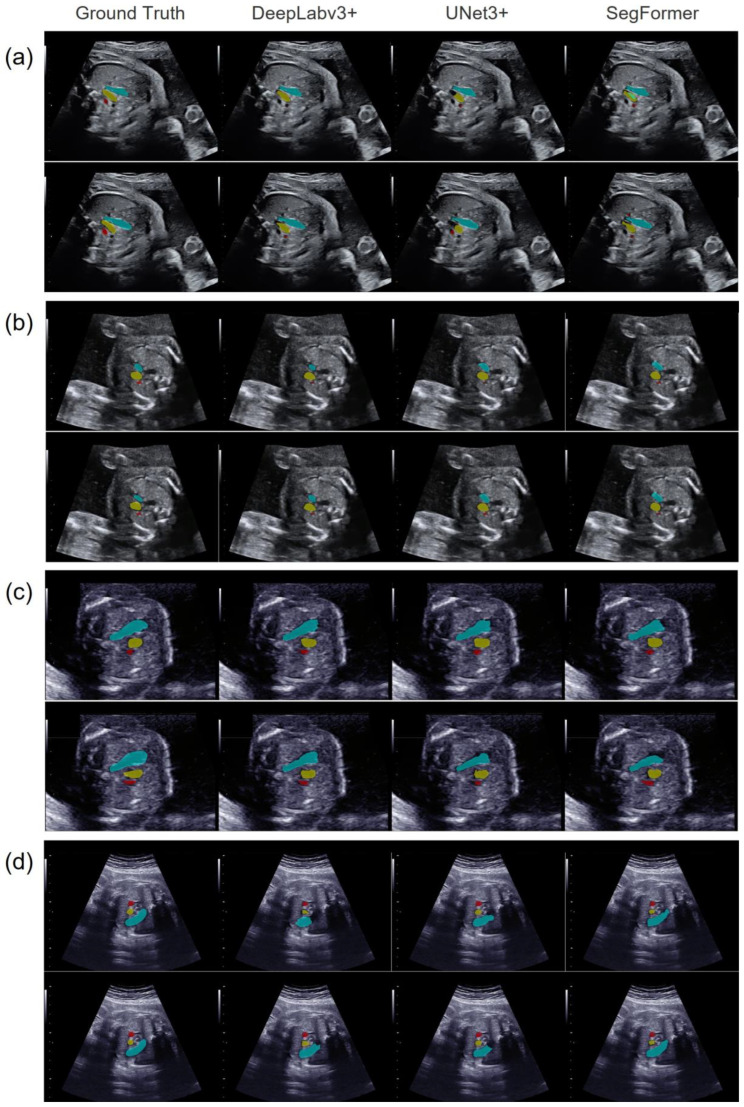
Representative vascular-segmentation images in the 3VV for the three models. The images on the far left contain annotated labels of the ground truth. One horizontal row presents the segmentation results for each model in the same case. (**a**) A normal case, (**b**) a CHD case with a low PA/Ao ratio, (**c**) a CHD case with a normal PA/Ao ratio, and (**d**) a CHD case with a high PA/Ao ratio. In each case, the upper images were manually extracted, while the lower images were automatically extracted using YOLOv7. The PA is shown in light blue, the Ao in yellow, and the SVC in red.

Figure 3 compares the segmentation results of the three models for (a) a normal case and three CHD cases: (b) tetralogy of Fallot (TOF), (c) atrial septal defect (ASD), and (d) coarctation of the aorta (with a ventricular septal defect). Pulmonary artery stenosis is a characteristic of TOF, in which the PA/Ao ratio is usually low. ASDs did not affect the morphology of the pulmonary artery and ascending aorta; therefore, the PA/Ao ratio was within the normal range in such cases. Owing to the narrowing of the ascending aorta in cases of CoA, the PA/Ao ratio is usually high. Thus, each case corresponded to a CHD group classified according to the PA/Ao ratio.

Table 1 compares the segmentation results of the three models obtained by manual and automated image extraction. In both the normal and CHD cases, the PA and Ao were segmented with comparable quality, whereas the segmentation of the SVC was inferior. SegFormer was superior for PA segmentation, and DeepLabv3+ was superior for Ao segmentation, regardless of whether manual or automated image extraction was performed.

### 3.2. Calculation and Assessment of the PA/Ao Ratio

Using the ground-truth labels of 270 normal cases obtained via manual image extraction, the ±2SD range of the PA/Ao ratio, 1.237 ± 0.364, was defined as the standard value. A comparison of the manual and automated image extraction methods revealed no significant differences in the PA/Ao ratios calculated using the ground-truth labels of normal cases (Supplementary Figure S3). Table 2 presents the PA/Ao ratios calculated for each method. Based on the standard values, DeepLabv3+ and UNet3+ were deemed suitable for normal cases. Furthermore, considering the classification of the normal and three CHD groups, YOLOv7 plus UNet3+ yielded the most accurate results, with the range of normal and CHD cases in the normal group matching and the PA/Ao ratios of the three CHD groups were in ascending order.

### 3.3. Screening Performance Using the PA/Ao Ratio

**Figure 4:**
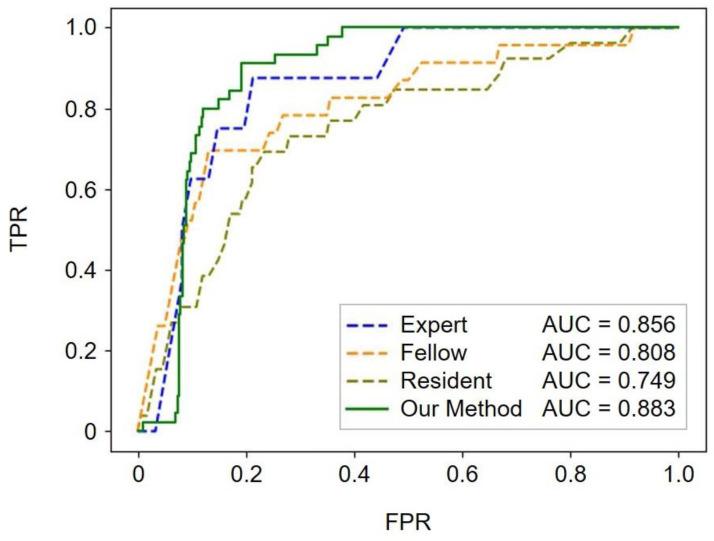
Performance of fetal cardiac ultrasound screening based on the PA/Ao ratio. The ROC curves illustrate the screening performances of experts, fellows, residents, and our automated method (YOLOv7 plus UNet3+). The mean values of the AUCs are reported in the legends. ROC, receiver operating characteristic; AUC, area under the receiver operating characteristic curve; TPR, true-positive rate; FPR, false-positive rate.

For ROC analysis of the performance of fetal cardiac ultrasound screening, the mean standard PA/Ao ratio was calculated as 1.237. In this screening performance comparison study, YOLOv7 plus UNet3+ was selected as the automated method as it yielded the best classification results, as presented in Table 2. As shown in Figure 4, our automated method achieved a mean AUC of 0.883, with AUCs of 0.749 for residents, 0.808 for fellows, and 0.856 for experts. Screening performance clearly depended on the examiners’ skill levels. The YOLOv7 plus UNet3+ algorithm performed better than the other algorithms, showing a slight edge over experts in terms of the mean AUC value; however, the TP rate was low when the FP rate was <0.1 in the ROC curve of the YOLOv7 plus UNet3+. Thus, the performance of our automated method was comparable to that of experts.

## 4. Discussion

Fetal cardiac ultrasound screening is generally recommended for all pregnancies [10,27]. However, manual scanning using an ultrasound probe causes differences in diagnostic techniques between experts and unskilled examiners and challenges image quality control [9]. Van Nisselrooij et al. previously conducted a retrospective quality assessment of fetal cardiac ultrasound images obtained during second-trimester screening in 92 cases of severe CHD. They reported significant differences between prenatally detected and undetected cases of CHD in terms of the adequate use of magnification and quality scores of the standard transverse scanning planes of the fetal heart [34]. To overcome these issues, many AI-based analyses of fetal ultrasound images have been developed for various uses, ranging from the anatomical detection of organs, such as the placenta, brain, and heart, to the measurement of different biometric parameters [16,35]. A framework for the multimodal analysis of ultrasound video images, eye movements, and ultrasound probe movements has also been proposed as a method of evaluating examiners’ skill levels during ultrasound scanning [36]. We previously developed an AI-based support system for fetal cardiac ultrasound screening to detect cardiac substructures in fetal ultrasound videos [29]. The AI-equipped software was approved as a medical device for clinical use in Japan by the Pharmaceuticals and Medical Devices Agency in July 2024 (approval number: 30600BZX00155000).

In the present study, we focused on the PA/Ao ratio in 3VV using AI-based image extraction and vascular segmentation to support unskilled examiners in the detection of CHD. There is currently a consensus on the guidelines that the PA/Ao ratio in 3VV is one of the representative biometric parameters to support the detection of CHD, particularly in those with outflow tract abnormalities [25]. In clinical practice, the vascular diameter is sometimes measured in cases in which abnormalities are strongly suspected, but it is not commonly measured in other cases owing to the significant time and effort required. In addition, the standard values have not yet been clearly defined. Wong et al. previously measured the PA/Ao ratio in 966 normal fetuses at 16–24 gestational weeks, reporting a mean ratio of 1.16 (median: 1.14, range: 0.61–1.86, 95% confidence interval: 0.87–1.58) [37]. Other reported mean values of the PA/Ao ratios for normal fetuses were 1.10 ± 0.09 [33], 1.119 ± 0.19 [38], 1.385 ± 0.154 [39], and 1.22 [40]. The standard values of the PA/Ao ratios vary little depending on gestational age [25,40]. According to the analysis of our dataset, 1.237 ± 0.364 was defined as the standard value of the PA/Ao ratio, which is similar to that of previous reports. PA is generally believed to be larger than Ao, yielding a PA/Ao ratio > 1. However, in our study, as in previous studies, the ratio was <1 for some normal fetuses. Therefore, Ao > PA does not necessarily indicate abnormalities. Because of this wide range of severity and forms of CHD, the PA/Ao ratio should be assessed in combination with other observations for CHD diagnosis [8]. Although the diagnostic accuracy cannot usually be explained by this index alone, we evaluated our automated method in terms of diagnostic accuracy in the present study.

Our automated method using YOLOv7 plus UNet3+ yielded the most appropriate classification of the normal and three CHD groups according to the PA/Ao ratio. Although the methods based on DeepLabv3+ or SegFormer performed better in vascular segmentation, their performance in group classification and the distinction between normal fetuses and those with CHD was inferior to that of YOLOv7 plus UNet3+. A possible reason for this is that the pretraining datasets of DeepLabv3+ and SegFormer did not include medical ultrasound images. In addition, many CHD cases had calculated PA/Ao ratios within the normal range, which may have reduced the performance of the algorithms in discriminating between normal and CHD.

Furthermore, in the comparison of the screening performance similar to that performed in clinical practice, the YOLOv7 plus UNet3+ algorithm outperformed both fellows and residents and achieved a level comparable to that of experts. A comparative screening performance study revealed that some examiners selected views other than the 3VV, complicating judgments of the 3VV. This was particularly evident in CHD cases and occurred more frequently with unskilled examiners. Images closer to 3VTV are often acquired and misinterpreted as 3VV. As the pulmonary artery moves to the ductus arteriosus in the cephalad direction, the maximum diameter of the PA may not be visible. Experts extracted the appropriate 3VV and accurately measured the vascular diameter in both the normal and CHD cases based on their experience. As many CHD cases generally show a normal PA/Ao ratio, the performance of our automated method is comparable to that of experts in the clinic. However, if the vascular structure is dramatically deformed in some CHD cases, automated 3VV extraction using vascular detection may be difficult.

Two related studies have recently been conducted. Yan et al. previously proposed a framework using YOLOv5 plus DeepLabv3 equipped with an attentional multiscale feature fusion module for detecting and segmenting three vessels in 3VV images [41]. Taksøe-Vester et al. developed a predictive, AI-assisted screening model to identify fetuses at risk of postnatal CoA by using automatic biometric measurements from the 4CV and 3VV during the 18–22-week scan. They used a U-Net architecture, while all cardiac measurements were performed using an AI model based on anatomical segmentation [42]. Both studies used still images, and the cardiac anatomical substructures were likely measured manually in the latter. In contrast, we propose a fully automated framework to extract 3VV images from fetal ultrasound videos and calculate the PA/Ao ratio based on vascular segmentation and programmed measurement of diameters.

### Limitations

First, a few fetal cardiac ultrasound videos are available, particularly for fetuses with CHD. For the automated extraction of images in 3VV, the confidence threshold for vascular detection using YOLOv7 was considerably lowered. We believe that better image extraction is possible by increasing the threshold and the number of videos. Second, our automated method may not respond to CHDs with drastically deformed vascular or cardiac structures. For clinical applications, different types of CHD need to be collected and examined. Third, the fetal ultrasound videos analyzed in the present study were obtained primarily by experts. Video quality varies depending on the skill level of the examiners, maternal body mass index, fetal position, fetal movement, amniotic fluid volume, and gestational weeks. These potential biases, image quality issues, and real-world performance issues can all affect the performance of our automated method. Therefore, domain shifts must be considered, while other technologies must be developed for the quality control of input images. Fourth, we used videos of ultrasonography machines from one vendor. For the clinical application of our AI-based automated system, its compatibility with other existing ultrasound equipment and potential cost barriers should be considered. Finally, the pulmonary artery and ascending aorta are located inside the thorax and can be cast in the shadows of the ribs and spine, or the contrast can be reduced owing to maternal obesity, complicating the acquisition of images with clear boundaries, which may result in under- or overestimation of their diameters. Therefore, the effects of shadows on the detection and measurement of the PA and Ao should be considered.

## 5. Conclusions

Herein, we developed an AI-based method for the automated and quantitative evaluation of 3VV images obtained during fetal cardiac ultrasound screening. To the best of our knowledge, this study is the first to demonstrate that our method may support unskilled examiners in the appropriate extraction of 3VV images and objective PA/Ao ratio calculations. For the clinical application of AI-based support systems in fetal cardiac ultrasound screening, core technologies need to be further strengthened, and feedback must be garnered from examiners and patients through performance evaluation tests and validation studies. Whether installed in ultrasound equipment or through cloud-based analysis, these automated biometric parameter estimation methods are expected to be integrated into clinical practice and will contribute to improving the total global prenatal diagnostic rate of CHD.

## Figures and Tables

**Table 1:** Results of segmentation evaluation of the three models via manual and automated image extraction using mDice.

		Normal Cases			CHD Cases		
Image Extraction	Segmentation	PA	Ao	SVC	PA	Ao	SVC
Manual	DeepLabv3+	0.812	0.822	0.677	0.686	0.733	0.547
	UNet3+	0.770	0.790	0.626	0.618	0.602	0.472
	SegFormer	0.812	0.714	0.667	0.699	0.702	0.555
Automated	DeepLabv3+	0.739	0.775	0.631	0.631	0.628	0.537
	UNet3+	0.713	0.707	0.541	0.612	0.608	0.492
	SegFormer	0.750	0.748	0.623	0.652	0.628	0.549

mDice, the mean values of the Dice coefficient.

**Table 2:** Evaluation of the PA/Ao ratio based on the vascular segmentation in the 3VV.

				CHD Cases	
Image Extraction	Segmentation	Normal Cases	Low	Normal	High
Manual	DeepLabv3+	1.200 ± 0.292	0.882 ± 0.334	1.228 ± 0.368	1.102 ± 0.167
	UNet3+	1.219 ± 0.281	0.967 ± 0.566	1.320 ± 0.362	1.396 ± 0.429
	SegFormer	1.306 ± 0.416	1.212 ± 0.382	1.228 ± 0.479	1.248 ± 0.279
Automated	DeepLabv3+	1.259 ± 0.360	1.051 ± 0.413	1.167 ± 0.391	1.311 ± 0.540
	UNet3+	1.227 ± 0.306	0.960 ± 0.309	1.251 ± 0.306	1.543 ± 0.611
	SegFormer	1.357 ± 0.493	1.065 ± 0.478	1.291 ± 0.461	1.643 ± 0.455

The values are the mean ± SD of each method. SD, standard deviation.

## Data Availability

Data are contained within the article and Supplementary Materials.

## Supplementary Materials

The following supporting information can be downloaded at https://www.mdpi.com/article/10.3390/bioengineering11121256/s1, Figure S1: Dataset and cross-validation; Figure S2: Procedures for examiners in the screening-performance comparison study; Figure S3: PA/Ao-ratio calculation using the ground-truth labels of normal cases; Table S1: Characteristics of the 35 fetuses with CHD in this study.
