# Supplementary material for: Automated Assessment of the Pulmonary Artery-to-Ascending Aorta Ratio in Fetal Cardiac Ultrasound Screening Using Artificial Intelligence
Source: Bioengineering (Basel). 2024 Dec 12;11(12):1256. doi: 10.3390/bioengineering11121256 (PMC11673077; doi:10.3390/bioengineering11121256)
Supplement: Supplementary file 1 [file bioengineering-11-01256-s001.zip › bioengineering-3332337-supplementary.pdf]

Supplementary information for

Automated Assessment of the Pulmonary Artery to the Ascending Aorta Ratio in Fetal Cardiac Ultrasound Screening Using Artificial Intelligence

Rina Aoyama, Masaaki Komatsu, Naoaki Harada, Reina Komatsu, Akira Sakai, Katsuji Takeda, Naoki Teraya, Ken Asada, Syuzo Kaneko, Kazuki Iwamoto, Ryu Matsuoka, Akihiko Sekizawa, Ryuji Hamamoto

The file contains

Supplementary Figures S1 – S3

Supplementary Table S1

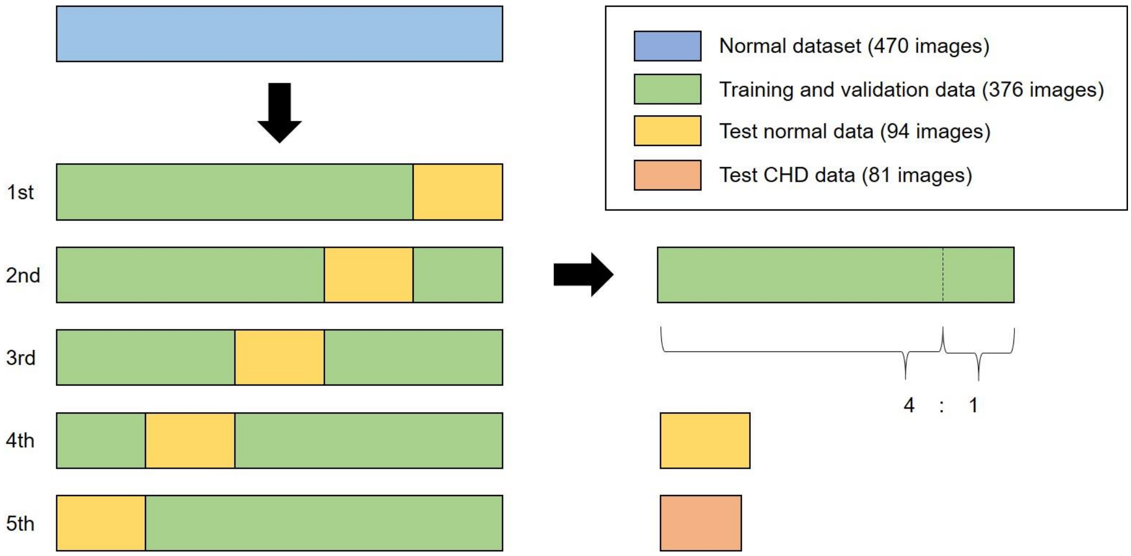

Supplementary Figure S1. Dataset and cross-validation. The dataset of 470 images obtained from 270 normal cases was cross-validated in five parts (5-fold cross validation). Training and validation data were automatically split 4:1. CHD, congenital heart disease.

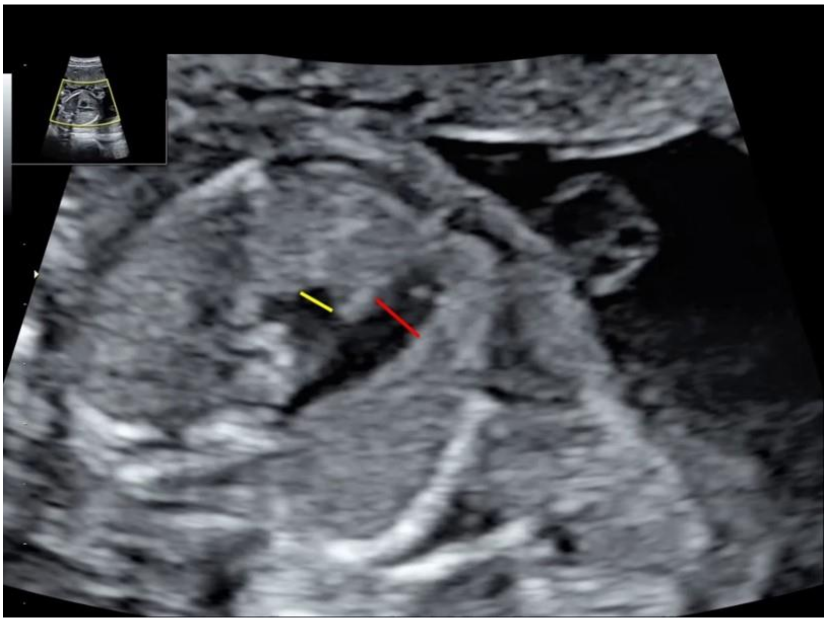

Supplementary Figure S2. Procedures for examiners in the screening performance comparison study. Examiners manually extracted 3VV images and drew on PA (red line) and Ao (yellow line) at points that could be considered as diameters. The program-based measurement of their lengths in pixels was automatically performed for the calculation of the PA/Ao ratio.

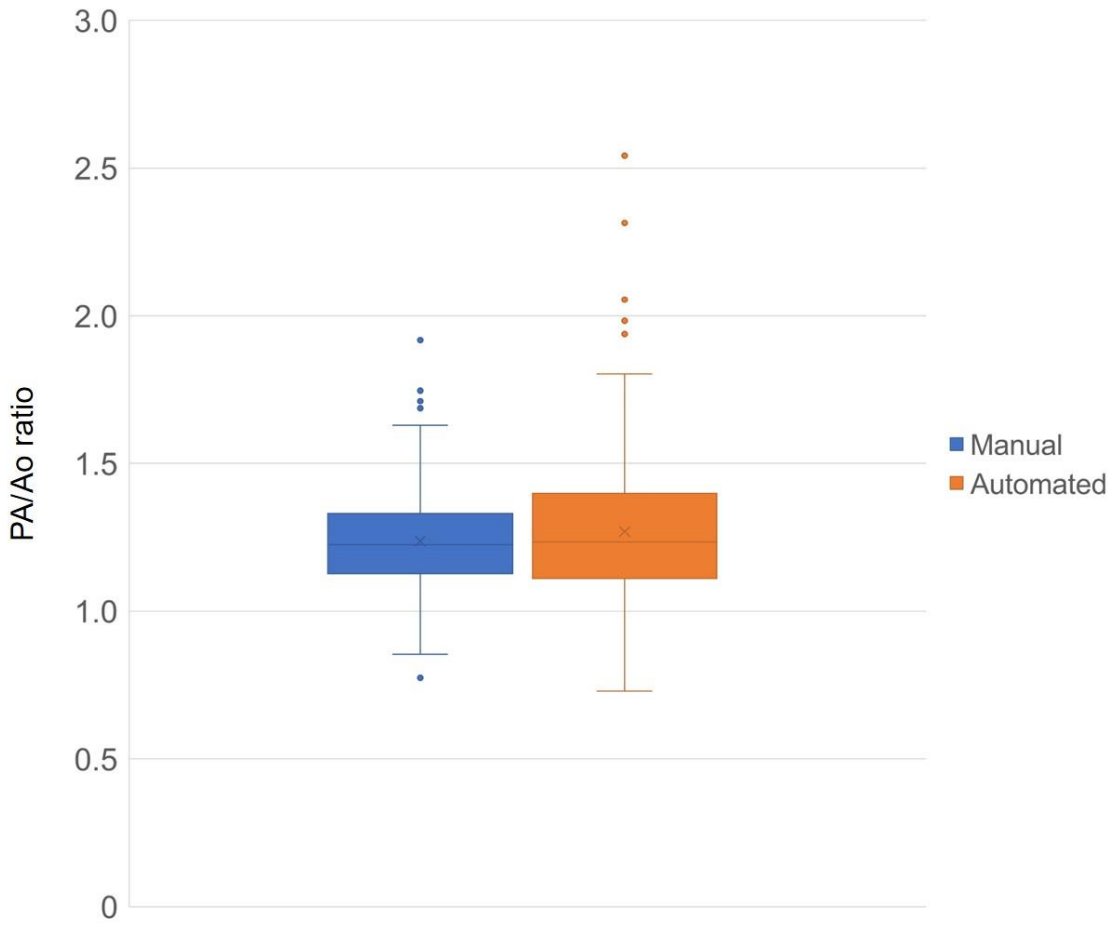

Supplementary Figure S3. The PA/Ao ratio calculation using the ground truth labels of normal cases. There were no significant differences between manual and automated image extraction methods (p = 0.095, Student's t-test).

CHD	Number of Cases
AVSD	4
TOF	4
PAIVS	3
TGA	2
DORV + VSD	2
Ebstein	2
PLSVC	2
DORV + VSD + PLSVC	1
DORV + TGA	1
DORV + TGA+CoA	1
DORV + TGA + VSD + pulmonary artery steonsis	1
ASD	1
VSD	1
CoA + VSD	1
TA	1
TA + pulmonary artery stenosis	1
Tricuspid insufficiency	1
PS	1
DAA	1
RAA	1
Trisomy 18	1
PAC	1
Bradycardia	1

Supplementary Table S1. Characteristics of the 35 CHD cases in this study. AVSD, atrioventricular septal defect; TOF, tetralogy of Fallot; PAIVS, pulmonary atresia with intact ventricular septum; TGA, transposition of the great arteries; DORV, double-outlet right ventricle; VSD, ventricular sepatal defect; PLSVC, persistent left superior vena cava; CoA, coarctation of aorta; ASD, atrial septal defect; TA, tricuspid atresia; PS, pulmonary valve stenosis; DAA, double aortic arch; RAA, right aortic arch; PAC, premature atrial contraction.
